# Expanding the scope of “trans-humanism”: situating within the framework of life and death education – the importance of a “trans-mystical mindset”

**DOI:** 10.3389/fpsyg.2024.1380665

**Published:** 2024-04-23

**Authors:** Huy P. Phan, Bing Hiong Ngu, Chao-Sheng Hsu, Si-Chi Chen, Lijuing Wu

**Affiliations:** ^1^School of Education, University of New England, Armidale, NSW, Australia; ^2^Department of Education, National Taipei University of Education, Taipei, Taiwan

**Keywords:** life and death education, trans-mystical mindset, transpersonalism, trans-mysticism, hierarchy of needs, mediative-reflective state, transcendence state, self-actualizing state

## Abstract

*Life* and *death education*, as noted from the literatures, has been studied and researched extensively in China, Malaysia, and Taiwan. Our own research undertakings over the past several years, situated in different sociocultural settings have delved into aspects of life and death that could help advance theoretical understanding of the subject matters (e.g., does the meaning of “effective life functioning” connote differing interpretations for different cultural groups?). Situating within the framework of life and death education, we expand the study of *trans-humanism* by introducing an extended prefix or nomenclature known as “trans-mystical”. Specifically, our philosophized concept of *trans-mysticism* considers a related concept, which we term as a “trans-mystical mindset”. A trans-mystical mindset, differing from an ordinary mindset, from our philosophical rationalization, is defined as “a person’s higher-order state of consciousness, espousing her perception, judgment, belief, and attempted interpretation of life and death phenomena that are mystifying and fall outside the ordinary boundaries of human psyche.” Our focus of inquiry, as reported in the present article, seeks to advance our proposition: that a trans-mystical mindset, unlike an ordinary mindset, may help a person to rationalize, appreciate, and understand metaphysical contexts, mystical experiences, and the like. This focus, interestingly, serves to highlight an important discourse - namely, that there is a dichotomy in theoretical lenses (i.e., objective reality vs. individual subjectivity) that a person may use to rationalize the significance or non-significance of universal contexts, events, phenomena, etc. (e.g., a person’s experience of “premonition”). As such, then, there is an important question that we seek to consider: whether philosophization, or the use of philosophical psychology, would yield perceived “scientific evidence” to support or to reject the study of metaphysicism, mysticism, and the like? For example, does our philosophization of an “equivalency” between a person’s trans-mystical mindset and her experience of self-transcendence help to normalize and/or to scientize the subject matters of metaphysicism, mysticism, etc.?

## 1 Introduction

The subject of *life* and *death education* (Chen, 2013; Huang, 2014; Seng and Lee, 2022) has significant daily relevance and applicability for us in society to consider. Personal understanding of *life education*, for example, may inform and educate a person about the diverse meanings and purposes of effective life functioning (e.g., the attainment of financial success in life vs. the attainment of good health). In relation to *death education*, likewise, a senior citizen’s spiritual knowledge of “transcendence” (Conn, 1998; Long, 2000; Ge and Yang, 2023), or his perceived “spiritual connectedness” to God (Laurin et al., 2014; Cohen-Zimerman et al., 2020), may assist him with his coping of grief. On a formal front, conceptual and/or empirical research development of life and death education may yield evidence to help elucidate and/or explain the underlying nature of the subject contents.

Our own teaching and research undertakings over the past decade have delved into different aspects of life and death education. For example, recently, we introduced a theoretical concept that we termed as a “holistic mindset”, or a person’s “multiple mindsets” (Phan et al., 2024). In brief, we theorize that a person may possess multiple contextual mindsets at *any moment in time* for adaptation and accommodation of different life and death contexts. For example, a Catholic nun may possess and exhibit a strong “spiritual mindset” whereas, in contrast, a scholar of Buddhism (Masel et al., 2012; Prude, 2019) may possess and exhibit a strong “philosophical mindset”. In a similar vein, a doctorate student preparing for an oral exam is more likely than most to possess and exhibit a “cognitive mindset”. Our theorization then, contends that a *specific life context* (e.g., the context of academic learning) would define and/or espouse a corresponding “contextual mindset”.

One particular life context that we are interested in is known as a “trans-mystical context” or a perceived mystical context. There are metaphysical or mystical life and death contexts in this world that are somewhat anomalistic and non-conventional. For example, some cultural groups may engage in the practice of “ancestor worshipping” (Steadman et al., 1996), whereas other cultural groups may view this practice with a sense of intellectual curiosity. In a similar vein, there are some of us who have reported the personal experience of “premonition”.^1^ We purport that ancestor worshipping, premonition, the belief in “reincarnation” (Nagaraj et al., 2013; Burley, 2014), and the like are metaphysical contexts or “non-daily” contexts. Such contexts, we contend, may elicit perceived experiences that are somewhat subjective and whether they fall outside the realm of objectivity and/or the realm of ordinary human psyche. Importantly, however, we reason that the *scientific premise of psychology* may provide sound, logical accounts to help explain the “uniqueness” of metaphysical contexts. That perhaps, despite individual subjectivity in perception, interpretation, and reason, metaphysical contexts and/or mystical experiences are just on par with “ordinary” contexts, life experiences, etc.

Our focus of inquiry for consideration relates to the advancement of the study of “trans-mysticism”. In particular, we philosophize a psychological concept, termed as a “trans-mystical mindset”, that may help to show how metaphysicism can be subjectively rational. In brief, we define a person’s “trans-mystical mindset” as:

A “contemplative higher-order, mystical” state that details his/her perception, attitude, judgment towards some “unknown” and/or extraordinary life concept, life phenomenon, event, situation, etc. (e.g., a person’s trans-mystical mindset towards the notion of “post-death” experience).

A trans-mystical mindset, as defined, may help a person to reason and/or to make meaningful sense of a metaphysical context and/or a mystical encounter (e.g., a person’s experience of premonition). By the same token, encountering a metaphysical context or a mystical life/death experience may help necessitate, facilitate, sustain a person’s trans-mystical mindset and his willingness to accept that such an encounter is valid. From this then, situating within the scope of life and death education (Chen, 2013; Huang, 2014; Seng and Lee, 2022), we premise a significant principle for consideration:

That metaphysical or mystical contexts (e.g., a perception of “spiritual transportation” to another time-space realm) are pivotal to the “formation” of a trans-mystical mindset, or that a person’s trans-mystical mindset is intimately linked to her trans-mystical life/death experiences.

Our position or standing is that psychological grounding (e.g., the use of the discourse of philosophical psychology, which entails the proposition of a trans-mystical mindset) may offer robust explanations for metaphysical encounters. More importantly, however, we contend that our philosophical undertaking here may form the basis for future examination of something that is somewhat contentious: that psychological inquiries alone cannot encapsulate and/or explain the uniqueness of metaphysical contexts; rather, as a possibility and something that is beyond the scope of this conceptual analysis article, is the fact that metaphysical contexts and mystical experiences have alternative interpretations and meanings – for example, the context of premonition (Cameron, 1958; Dossey, 2009), one’s belief in reincarnation (Nagaraj et al., 2013; Burley, 2014), and the like cannot simply be validated or vindicated by scientific inquiries.

Overall, then, the present conceptual analysis article uses *philosophical psychology* (Thagard, 2014; Thagard, 2018; Phan et al., 2024) to help “normalize” and/or to “scientize” the subject matters of trans-mysticism. That philosophizing the concept of trans-mysticism (e.g., a trans-mystical mindset) and “benchmarking” this against Maslow’s (1968, 1969) theory of the “hierarchy of needs” may, in fact, validate and/or legitimize the importance of metaphysical contexts, mystical life and death experiences, etc. This line of inquiry, we contend, emphasizes an important standing: the premise of “objectivity” vs. the premise of “subjectivity”. Objectivity indicates *consistency*, *transparency*, *realism*, and *non-biased judgment*, whereas subjectivity, in contrast, considers *openness*, *personal viewpoint* and *interpretation*, and *individualistic thinking*. Regardless of one’s position, we firmly believe that our philosophized concept of trans-mysticism and thereafter may advance the study of life and death education. In the following section of the article, we discuss a number of elements – namely:

- An introduction of a theoretical account of the subject of life and death education.
- An examination of the nature of a proposed life and death-related concept that we term as “trans-mysticism”.
- A proposition of a theoretical premise, which purports the process of transformation of a person’s ordinary mindset, resulting in a trans-mystical mindset.
- A proposition of an association, which purports a situational placement or contextualization, highlighting a potential equivalence between a trans-mystical mindset and a state of self-transcendence.
- A discussion of a few notable inquiries for teaching and research development purposes.

## 2 The importance of life and death education: a brief introduction

*Life* and *death education* (Chen, 2013; Huang, 2014; Seng and Lee, 2022) is an interesting subject for teaching and learning, given its potential relevance and significance for daily life purposes. The study of life and death education, in its entirety, seeks to understand and appreciate the intricacies or complexities of human existence from different historical-sociocultural perspectives (e.g., what does proactive life functioning mean for South Pacific Islanders?). *Life education*, in brief, relates to the teaching of specific tenets about life that may enable and/or assist a person to live a cherished and self-fulfilling life. A cherished and self-fulfilling life, say, may consist of a person’s feeling of self-gratification, arising from her successful attainment of financial wealth. In a similar vein, but somewhat different, a cherished and self-fulfilling life may reflect a person’s daily practice to impart his life wisdom onto others. Such teaching is meaningful and may serve to enlighten individuals, their families, and society in general. One distinction, in this case, refers to a person’s appreciation and acknowledgment that variations in historical-sociocultural context (e.g., a child who grows up in an Indonesian historical-sociocultural context) give rise to different life courses and life trajectories (e.g., a child who grows up in an Indonesian sociocultural context, and the shaping of her aspirations, desires, future intentions, etc.). In a similar vein, a person’s life wisdom or life knowledge (Goldstein and Kornfield, 1987, Sternberg and Glück, 2019, Chattopadhyay, 2022) may be transformed into practice for daily life purposes.

Life education seeks to provide quality teaching, theoretical insights, and relevant information that may assist, explain, and facilitate proactive daily life functioning. Proactive life functioning on a daily basis is vibrant and healthy, helping a person to fulfill and attain a desirable life trajectory or trajectories. Different life contexts (e.g., the context of academic learning) on a daily basis, we contend, connote different types of proactive functioning – for example, the life context of awareness of the danger that COVID-19 poses (Willyard, 2023) may compel a person, in this case, to seek appropriate pathways to ensure that she has a healthy life trajectory. In a similar vein, the life context of the importance of academic attainments may shape a student’s mindset to seek mastery and deep, meaningful learning experiences in his schooling. Regardless of diversity of life contexts, life education places emphasis on the recognition, promotion, and development of a cherished and self-fulfilling life.

*Death education*, or the *study of thanatology* (Meagher and Balk, 2013; Chapple et al., 2017), in contrast, seeks to understand the intricate nature of death and other dying-related matters (e.g., the process of grief for a loved one). For example, angst, stress, sadness, and depression are life matters that closely associate with death. Unlike life education, which is positive, vibrant, and self-fulfilling, death education is morbid and undesirable for teaching and learning. For example, the teaching of death education seeks to educate individuals, family members, and society the following aspects:

- The perception, viewpoint, and/or belief that one has towards the subject of death (e.g., how does one feel, at present, knowing that a loved one is facing a critical illness?).
- Personal care and preparation from others (e.g., social workers, volunteers) to assist with the impending encounter and/or facing of death.
- Stages and processes (e.g., counselling, spiritual advice, etc.) that are associated with grief and bereavement upon the death of a loved on.
- Consideration of programs, strategies, pathways, etc. that could help alleviate the negative emotions, feelings, perceptions, etc. that one may have when faced with a death-related matter.

Our study of life and death education for teaching and theoretical contribution purposes over the past decade has led us to undertake a few notable developments – namely, the testament of the following: Focus on instructional designs and pedagogical approaches (2.1), Research inquiries for consideration (2.2), and Advancement in theoretical contributions (2.3).

### 2.1 Focus on instructional designs and pedagogical approaches

Focus on appropriate instructional designs and pedagogical approaches that may instill appreciation and facilitate effective learning experiences for the subject life and death education (e.g., appreciating that death education has potential daily life relevance). We propose an interesting idea known as “theoretical infusion”, which involves the practice of “infusion” of a particular faith, epistemological belief, customary practice, discourse, etc. in the teaching of life and death. “Spiritual infusion”, for example, details the incorporation of spirituality, or one’s spiritual faith (Schneiders, 1986; Wagani and Colucci, 2018; Villani et al., 2019), to complement the teaching of life and death, making it more stimulating and “life-related” for learning. Theoretical infusion (e.g., Buddhist spiritual infusion), we contend, may serve to associate subject contents of life and death with other meaningful and/or related contents. In other words, theoretical infusion is used to encourage students to appreciate subject contents of other topics and/or subjects (e.g., appreciating the importance of Christianity from a life perspective) within the context of life and death education. By the same token, we rationalize the benefits of embedding subject contents of life and death within other subject contexts (e.g., how does Christian faith view death?). Having said this, however, we also acknowledge an important mentioning from one of our reviewers in an earlier draft of this article – that we need to also consider the potential “negativity” of our idea of theoretical infusion. That engaging in theoretical infusion (e.g., infusing a particular religious or spiritual faith to support the teaching of death education) may, in fact, amount to and/or be perceived as a form of “indoctrination”. A student with no religious affiliation, in this instance, may feel pressured to accept the practice of “Buddhist spiritual infusion” as a “norm”.

Aside from theoretical infusion, we also propose and use another discourse that we term as “active transformation”. In brief, active transformation relates to one’s self-cognizance of daily practicality of knowledge pertaining to life and death. In other words, active transformation emphasizes the important nexus between theory and practice – for example, how can a teenager use her personal understanding of Confucianism (Yao, 2000; Havens, 2013) to assist others in the neighborhood? As such, then, we rationalize that our idea or theoretical premise of active transformation may serve to impart benefits for individuals and society. For example, a mother may accompany her son and make weekly visits, offering spiritual advice and life wisdom on different life and death-related matters to those in this need. This voluntary periodic engagement reflects her willingness to help others in the community and, more importantly, showcases proactive practice of active transformation of life wisdom, or life knowledge. Again, having said this, we are cognizant of one of our reviewers’ earlier mentioning: that the idea or the theoretical premise of active transformation may, likewise, produce negative yields. A person’s inclination towards some form of negativity, in this case, may compel her to engage in negative or maladaptive functioning. That rather than offering sound spiritual advice, a mother may instead transform her life wisdom about spirituality for negative purposes (e.g., a purposively act to indoctrinate a senior citizen with a biased view of Buddhist spirituality).

### 2.2 Research inquiries for consideration

Concerted attempts to seek new research frontiers that may amplify the importance of the subject life and death. One aspect of our research development, at present, seeks to understand and appreciate the importance of life and death from two contrasting positions: *objectivity* and *subjectivity*. Certain life and death matters (e.g., the proposed notion of “post-death” experience) (Phan et al., 2024), we contend, compel and/or require us to seek alternative research discourses for understanding. For example, over the past few years, our use of philosophical reasoning (Thagard, 2014; Thagard, 2018; Phan et al., 2024) has assisted us to understand about the study of life and death experiences (e.g., attainment of theoretical insights and explanatory accounts of life and death). Philosophical inquiries, from our point of view, may to complement contrasting research discourses and help to yield scientific credence for support. Engaging in philosophical analysis, we contend, may serve to encourage researchers to think non-conventionally and outside the box. Higher-order thinking, reflection, etc. may give rise to contemplation of research propositions for discussion. Our intent over the past several years has been to expand the scope of life and death education (Chen, 2013; Huang, 2014; Seng and Lee, 2022) by seeking to understand the known and unknown “unknowns” of life and death experiences. This line of research development is somewhat different from other inquiries and research undertakings that place emphasis on the “knowns” of life and death experiences (e.g., the intimate process of grief). The “unknowns” of life and death are more interesting as they delve into unexplained complexities of human existence that do not have clear, consistent explanatory accounts.

### 2.3 Advancement in theoretical contributions

Our interest, aside from teaching and research purposes, also seeks to make meaningful theoretical contributions to the study of life and death education (Chen, 2013, Huang, 2014, Seng and Lee, 2022). One aspect of our research development focuses on the examination and reading of the literatures, pertaining to the importance of *variations* of different historical-sociocultural contexts of life and death functioning. In brief, we note from our own research undertakings that different historical-sociocultural contexts offer unique insights into the viewpoint, opinion, perception, and interpretation of life and death experiences. For example, in terms of life functioning, we note that many Taiwanese believe in the attainment of “spiritual growth” in place of financial wealth. In a similar vein, many Taiwanese engage in the practice of “ancestor worshipping” (Steadman et al., 1996) and believe in the “afterlife” (Segal, 2004; Jones, 2016).

Gauging into the “historical-sociocultural contextualization” of life and death is meaningful as it offers unique understandings of life and death experiences. One distinction about this focus of inquiry is that unlike other disciplines and/or fields of research, the subject of life and death has comparable and contrasting viewpoints, opinions, perceptions, interpretations, etc. That understanding of life and/or of death (e.g., is there any validity to the notion of afterlife?), for example, differs for different ethnic-cultural groups. At present, one of our research undertakings seeks to understand the uniqueness of the Australian Aboriginal and Torres Strait Islander culture and her viewpoint, interpretation, status quo, etc. about life and death. One of our colleagues, who is a Torres Strait Islander, has shared with us some interesting facts for consideration. According to our colleague, many Australian Aboriginal and Torres Strait Islander peoples believe in the existence of rebirth where a deceased is transformed into a new “being”. To facilitate success in such a process, it is poignant that relatives and loved ones do not mention the deceased’s name for 12 months [e.g., “Do you remember when Sarah (i.e., the deceased) used to say this…?”].

## 3 The present conceptualization

Our aforementioned description of life and death education (Chen, 2013; Huang, 2014; Seng and Lee, 2022) has provided grounding for our philosophical inquiry and research undertaking, which delve into the nature of a proposed concept known as “trans-mysticism” or, alternatively, trans-mystical studies. For us, as a proposition, trans-mysticism is a combination or the unification of two *distinct areas of research of trans-humanism: transpersonalism* (Strohl, 1998; Lancaster and Linders, 2019) and *mysticism* (Schneiderman, 1967; Bronkhorst, 2022). It is important to note that our proposed term of trans-humanism differs from the more recent practice or use of the term (i.e., “trans-humanism”), which contends the possibility that we could use technological advances to augment human capabilities. Trans-mysticism, for us specifically, is a psychological premise that that may assist researchers, educators, students, etc. to understand, appreciate, and/or accept the existence of metaphysical contexts and the anomalistic and “non-realistic states” of life and death. More importantly, we rationalize that our philosophized concept of trans-mysticism (e.g., a “trans-mystical mindset”) may help to “normalize” and/or to “scientize” the subject matters of metaphysicism, mysticism, and the like. For example, one of our articles published recently (Phan et al., 2021) introduces readers to a specific cultural belief (and/or the cultural practice) known as the “underworld” or the “other world” by which a person could travel to interact with loved ones who have moved on. This mentioning may, indeed, give rise to criticisms, disbeliefs, doubts, uncertainties, etc. In a similar vein, unbeknown to some or many in the Western world, perhaps, but the cultural practice of ancestor worshipping (Townsend, 1969; Steadman et al., 1996; Lakos, 2010; Clark and Palmer, 2016) connotes a specific meaning for those in the Eastern world. Aside from veneration for the dead, this cultural practice also signifies the importance in what is known as “spiritual connectedness” or spiritual communication between the dead and the living – for instance, a daughter may pay homage to her deceased father by lighting incenses and asking for his specific blessing to assist her with the forthcoming final exams.

We reason and contend that philosophical research inquiries in the social sciences (i.e., a research inquiry that utilizes the discourse of philosophical psychology) may affirm one of two things: validating a proposed inquiry with supporting “philosophical” evidence *or* invalidating a proposed inquiry due to a lack of “philosophical” evidence – for example: that there is support for the proposed concept of trans-mysticism, which may help to provide robust explanations for metaphysical encounters. Of course, it is plausible to purport that trans-mysticism may simply be philosophical and lacks logical credence or legitimate merits for further consideration. One of our reviewers, in an earlier draft of the manuscript, offered an interesting critique: that resorting to the use of philosophical psychology (Thagard, 2014; Thagard, 2018; Phan et al., 2024) or that philosophizing about the nature of a metaphysical context (e.g., one’s ability to interact with a loved one who has moved on) does not necessarily make it valid or credible for research development. Our conceptualized approach, in this case, argues that psychological tenets may be used to explain the underlying nature of metaphysical contexts and/or mystical experiences in life. That the psychological concept of trans-mysticism may, for example:

- Help to “normalize”, “scientize”, and/or “legitimize” the study of metaphysical contexts and/or non-ordinary or extraordinary realms of human existence (Rush, 2011; Pasi, 2015).
- Help us appreciate the trans-mystical nature of metaphysical contexts and/or mystical experiences (e.g., a person’s testament of her ability to “detect” dark spiritual “energy” of a loved one).

Over the course of our research development, from conception to subsequent refinement of the article, we have evolved in our thinking and deliberation. Poignant then is our main focus of inquiry, which seeks to capitalize on the use of psychological theories (e.g., transpersonalism) to explain the intricate nature of metaphysicism, mysticism, and the like. Central to our thesis is the robust explanatory account, epistemically objective in nature, of the aforementioned subject of one’s metaphysical or mystical encounters. That ultimately, perhaps, differing subjective universal encounters and/or experiences (e.g., the metaphysical encounter of a loved one who has moved on vs. the daily encounter of a next-door neighbor) may “subsume” within a common prism or theoretical lens for understanding. A related inquiry for future consideration, which falls outside the scope of the present article relates to the confirmation or the epistemic validation of the trans-mystical nature of metaphysical contexts and/or mystical experiences (e.g., that indeed there is something unique or mysterious about a particular metaphysical encounter, and this personal experience does not coincide with objective reality).

### 3.1 A brief account of transpersonalism and transpersonal psychology

In this section of the article, we briefly discuss a related topic known as *transpersonalism* (Strohl, 1998; Lancaster and Linders, 2019) and *transpersonal psychology* (Maslow, 1969; Hartelius et al., 2007). This topic, we contend, is important and relates to our theoretical premise of trans-mysticism. It is interesting to note that there is a distinction between transpersonalism and transpersonal psychology or that, in fact, the two areas or disciplines are not identical or equivalent (Friedman, 2002; Shorrock, 2008). Friedman’s (2002) theoretical account offers a detailed analysis – for example:

“The former [i.e., transpersonalism] is a broadly defined domain of inquiry that can legitimately include a diversity of methods ranging from those of the humanities to those of a variety of scientific endeavors. Psychology, on the other hand, is defined by most psychologists as a scientific discipline; except for a few humanistic and transpersonal adherents who insist that including alternative, that is, nonscientific, approaches is important for the discipline, science is widely accepted as the mainstay of the discipline…. Furthermore, I see transpersonal psychology foremost as a field within the discipline of scientific psychology that focuses on those aspects of trans personal studies that involve the individual, including thoughts, feelings, and behaviors as found in the individual’s biological, cultural, social, and wider contexts” (pp. 180–181).

A more detailed explanation is noted in Shorrock’s (2008) book, titled “*The Transpersonal in Psychology, Psychotherapy, and Counselling*”. Shorrock’s (2008) account of transpersonalism and transpersonal psychology is comprehensive, outlining the genesis, complexity, and the numerous definitions and viewpoints that scholars over the years have proposed. The word count of the present article limits us from detailing Shorrock’s (2008) book and/or the complete gamut of definitions, viewpoints, perspectives, etc. of both disciplines. For the purpose of our rationale, we provide a few definitions of the two areas/disciplines for readers to appreciate (Table 1). From Table 1, a point of commonality between transpersonal psychology (Tart, 1975; Lajoie and Shapiro, 1992; Cunningham, 2007) and transpersonalism (Strohl, 1998; Lancaster and Linders, 2019), in this case, is the use of the prefix “trans” (Lancaster and Linders, 2019) or the extended prefix or term “transpersonal”, which is defined as “as reaching beyond the personal realm or transcending the singular, personal state of being” (Clark, 2016). Moreover, from our analysis, the significance or the uniqueness of transpersonalism and transpersonal psychology relates to the following: that decisions to accept or to reject transpersonalism and/or transpersonal psychology are largely based on scientific rigor and a researcher’s ability to empirically validate using scientific means (e.g., is it possible?) (Friedman, 2002; Shorrock, 2008). Transpersonal psychology is considered as being more robust, valid, and/or legitimate for its scope, which closely aligns to the rigor of scientific psychology (Friedman, 2002; Shorrock, 2008).

**Table 1:** A summary of sample definitions of transpersonalism and transpersonal psychology.

Authors	Transpersonal psychology	Authors	Transpersonalism
Cunningham (2007)	“The term “transpersonal psychology” is used differently by different theorists. As a result, the content of transpersonal psychology has come to mean different things to different people” (p. 42). “The broad definitional themes of transpersonal psychology – ‘highest or ultimate potential’, ‘phenomena beyond the ego’, ‘human transformation and transcendence’, ‘transcendent states of consciousness’, ‘psychospiritual development’, ‘integrative/holistic psychology’ – may all sound quite esoteric, but they refer to highly practical experiences and behaviors” (p. 45).	Clark (2016)	“Transpersonalism is not a religion or spiritual practice, though issues of spirituality and spiritual experience are at the forefront of the TP [i.e., transpersonal psychology] movement (Daniels, 2002). Transpersonalism is not ‘new age’, though it does represent a paradigm shift in thought, science, culture, and consciousness. While metaphysics are important in TP, multiple ways of knowing are also honored as one considers intuition, contemplation, and integrative awareness (Daniels, 2002). TP provides us with a way of considering how we can learn to care deeply, creating nursing interactions that are meaningful for both the patient and the nurse” (p. 3).
Lajoie and Shapiro (1992)	“Transpersonal psychology is concerned with the study of humanity’s highest potential, and with the recognition, understanding, and realization of unitive, spiritual, and transcendent states of consciousness” (p. 91).	Lancaster and Linders (2019)	“Transpersonalism concerns an expanded perspective of human identity and potential which affects all areas of human endeavor and participation” (p. 40). “Transpersonalism extends beyond researching the nature of self and wider questions about the mind. It attracts those who are questing for a more enriched way of being, as was traditionally connoted in religious societies by terms such as ‘soteriology’, ‘enlightenment’, or ‘union with God’. A central issue for transpersonalism today concerns the worldview within which such transformation is situated: Do we include non-material realms, a divine or transcendent component, forever beyond the lens of scientific exploration? Or can we incorporate in an extended scientific paradigm notions of transcendence that are explicable in terms of non-religious categories?” (p. 41).
Tart (1975)	“Transpersonal psychology is the title given to an emerging force in the psychology field by a group of psychologists and professional men and women from other fields who are interested in those *ultimate* human capacities and potentialities that have no systematic place in positivistic or behavioristic theory (‘first force’), classical psychoanalytic theory (‘second force’), or humanistic psychology (‘third force’). The emerging Transpersonal Psychology (‘fourth force’) is concerned specifically with the *empirical*, scientific study of, and responsible implementation of the findings relevant to, becoming, individual and species-wide meta-needs, ultimate values, unitive consciousness, peak experiences, B-values, ecstasy, mystical experience, awe, being, self-actualization, essence, bliss, wonder, ultimate meaning, transcendence of the self, spirit, oneness, cosmic awareness, individual and species-wide synergy, maximal interpersonal encounter, sacralization of everyday life, transcendental phenomena, cosmic self humor and playfulness, maximal sensory awareness, responsiveness and expression, and related concepts, experiences, and activities” (p. 2).	Strohl (1998)	“Transpersonalism is an approach that does not challenge or supplant other models, but it respectfully considers an expanded view of human nature wile incorporating elements of behaviorism, psychoanalysis, humanistic psychology, Jungian analysis, and Eastern psychology. Transpersonalism’s distinction from other models rests on transpersonalism having a fundamental difference in its philosophical worldview (McDermott, 1993)” (p. 399).

Despite contrasting definitions (Table 1), we argue that both transpersonalism (Strohl, 1998, Lancaster and Linders, 2019) and transpersonal psychology (Tart, 1975, Lajoie and Shapiro, 1992, Cunningham, 2007) are comparable with each other in terms of interpretation, understanding, and inference. Central to this rationale is that regardless of methodological considerations (e.g., what methodological approach would be best to investigate…?), the study of transpersonalism and the study of transpersonal psychology both subsume within or fall under the umbrella of what we term as “trans-humanistic” development. That trans-humanistic studies, in their entirety, can offer insights and theoretical understandings into the underlying nature of “humanism”. Moreover, we premise that our philosophized nomenclature and/or concept of trans-mysticism, likewise, may subsume within the overarching framework of trans-humanism. In other words, for consideration, is the following extrapolation: that the trans-humanistic framework, especially the tenets of trans-mysticism may provide theoretical grounding to help us gauge into the *logic*, *validity*, and *legitimacy* (e.g., objectivity vs. subjectivity) of the study of metaphysical contexts and mystical life and death experiences – for example, near-death experiences, spiritually transformative experiences, spiritual awakenings, peak experiences, and ecstatic experiences.

### 3.2 Trans-mystical development: a proposition

*Trans-mysticism*, as a distinct concept, may contribute to the study of life and death education (Chen, 2013; Huang, 2014; Seng and Lee, 2022) by accentuating the significance of metaphysical contexts and mystical of life and death experiences, such as:

- The personal experience of “premonition” (Cameron, 1958; Dossey, 2009).
- Personal belief in the concept of “reincarnation” (Nagaraj et al., 2013; Barua, 2017), or the concept of the endless cycle of “birth-death-rebirth”.
- The personal experience of “spirit communication” with loved ones who have moved on (e.g., the ritual practice of Guan Lou Yin) (Buckland, 2004; Phan et al., 2021).
- The personal experience of “time–space transcendence” (i.e., one’s ability to transcend to another time–space context) (Phan et al., 2024).

The proposed prefix or nomenclature “trans-mystical” is somewhat unique for its unification of two distinct areas of research: trans-humanistic studies (e.g., the study of a person’s experience of self-transcendence, which showcases a higher-order form of life functioning) + mystical studies (e.g., the study of a person’s esoteric experience of perceived spirit communication). Trans-mysticism, in accordance with our rationale, is closely associated with the specific *subject matters* of metaphysicism and mysticism. That our justification for the inclusion of the concept of trans-mysticism arises from the following understanding: *that there is an intimate association between life/death context (i.e., metaphysical or mystical context) and a person’s individual mindset*. Moreover, from our point of view, the theoretical premise of psychological concept of trans-mysticism is as follows: that personal experience of metaphysical contexts and/or mystical phenomena may give rise to the *necessitation*, *development*, and *manifestation* of a “trans-mystical mindset”. What is a trans-mystical mindset, which subsumes under the theoretical framework of trans-mysticism? For the context of the present article, we define a trans-mystical mindset as:

The ultimate human experience and/or a higher-order state of consciousness of a person, espousing her *perception*, *judgment*, *belief*, and *attempted interpretation* of metaphysical contexts and/or of life and death phenomena that are mystifying and fall outside the ordinary boundaries of human psyche.

**Figure 1:**
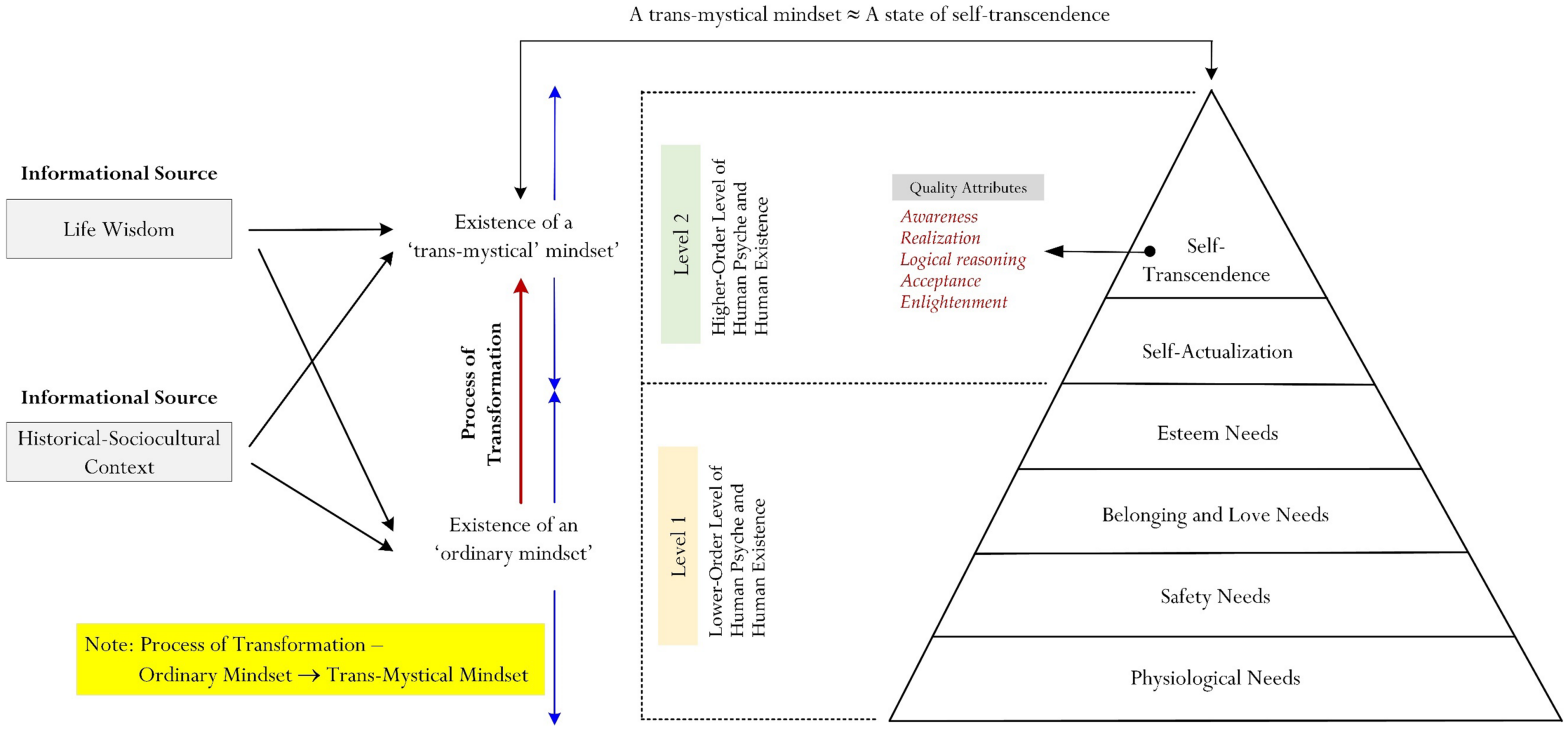
Structure of trans-mystical mindset.

Our philosophization contends that a trans-mystical mindset is *contextual* (i.e., it is contextualized or is situated within the metaphysical or the trans-mystical life and death contexts) and differs, in this case, from a person’s “ordinary” mindset (Figure 1). There are perhaps a few unique characteristics for us to consider – namely:

- A trans-mystical mindset is an internalized state that is perceived as being complex and/or higher-order. A trans-mystical mindset is different from an ordinary mindset, which espouses the “perception of normality” or the “realm of conventional human psyche”. An ordinary mindset, in this case, manifests and functions to facilitate successful adaptation of typical or standard daily life contexts (e.g., the context of academic learning in university or the context of a bank employee adapting to his new workplace environment).
- Existence of a trans-mystical mindset corresponds to and/or contextualizes to a specific metaphysical context, which may result in a person experiencing some form of mysticism (e.g., a person’s experience of premonition).
- There is a demarcation between what is “ordinary” and what is “extraordinary” and this distinction, in fact, explains the nature between an ordinary mindset and a trans-mystical mindset (Figure 1).

Our philosophization has so far introduced an interesting discourse for consideration: the *uniqueness* in perception, interpretation, and understanding of life and death (i.e., the perspective of objectivity vs. the perspective of subjectivity). The *perspective of objectivity* (Hanfstingl, 2022) emphasizes the importance of impartiality, unbiased interpretation and logical judgment, and the use of facts and verifiable evidence. For example, in terms of “negativity”, poverty, suffering, uncertainty, despair, and confusion (Zhang, 2003; McCartney et al., 2007; Mistry et al., 2009) are attributes that many of us experience on a daily basis. Natural tendency, in this case, would dictate that one’s personal mindset seeks out opportunities, pathways, means, etc. to help rectify or resolve such negative life experiences. The *perspective of subjectivity* (Lundberg et al., 2023), in contrast, emphasizes individualism, a person’s own sense of interpretation and point of view, and potentially biased judgment. For example, a person’s feeling and subjective interpretation may give rise to her conviction and insistence that spirit communication (Buckland, 2004), premonition (Cameron, 1958), time–space displacement, and the like are trans-mystical experiences that do not coincide with everyday objective reality. Subjectivity, in this sense, may associate with what we refer to as “subjective rationality” or “subjective rationalization”. It is interesting to note that unlike objectivity, subjective rationality may reflect and/or encompass the uniqueness of what we term as “social and/or cultural mediation”. That particular culture (e.g., Taiwanese culture), in this instance, may convey and/or mediate messages of acceptance, appropriateness, etc. of metaphysical experiences (e.g., premonition).

### 3.3 Ordinary mindset, trans-mystical mindset, and self-transcendence

An interesting position is that it is plausible to approach the study of metaphysicism and mysticism from a psychological point of view. There are in this sense several notable inquiries relating to the study of life and death education that are somewhat unique but, importantly, we are not able to address and/or answer here. Central to our thesis, as previously mentioned, is the use of philosophical psychology (e.g., the proposition of a trans-mystical mindset) to help normalize and/or to scientize the subject matters of metaphysicism and mysticism. Beyond the scope of our examination and something that is more contentious, perhaps, is the potential study of the epistemic validation of the underlying nature of metaphysicism – that, indeed, there is something *mysterious* about metaphysical contexts and that these do not coincide with the realm of ordinary boundaries (e.g., that the personal experience of premonition). In a similar vein, this mentioning of the “mystique” of metaphysicism raises several questions for future research to consider:

- Is it a case of subjective rationalization or subjective rationality – that the *perception* of mystique of metaphysical contexts is subjective and individual and not universal in terms of rationalization (e.g., that a person’s subjective rationalization of metaphysical contexts does not necessarily hold for another person)?
- Is it beyond the scientific confines and/or the scientific rigor of psychology, as a distinct field of research, and that some alternative epistemology is required in order for us to study the complexity of metaphysicism?
- Is it a valid discourse for us to suggest that there is scientific credence to study the epistemic validation or invalidation of metaphysicism?

The present study context considers an interesting premise: that psychological understanding, situated within the boundary of realistic objectivity, may help explain the nature of metaphysicism and mysticism. In other words, a trans-mystical mindset, psychological in makeup, may assist a person to *accommodate*, *adapt*, *resolve*, and *interpret* the intricacies of metaphysical contexts. That indeed, from our considered viewpoint, metaphysical contexts (e.g., a daughter’s experience of spirit communication with her loved ones) do not necessarily differ from daily life contexts (e.g., a teenager’s romantic feeling for his classmate). Individual differences (e.g., a person’s insistence that he has reincarnated), in this sense, are perhaps subjective – that subjective rationalization is prevalent and may serve to attribute to one’s own conviction of a metaphysical experience.

Unlike an ordinary or a normal mindset, a trans-mystical mindset does not simply eventuate. It is not automatic, spontaneous, and/or instantaneous. Rather, the perceived “unknowns” of this world, or a specific unknown context that one may confront at a particular moment in time, may initiate and stimulate a trans-mystical mindset. Our philosophization contends that a trans-mystical mindset reflects a person’s experience of being able to “transcend” herself from an ordinary level of human psyche to an expansive, extraordinary level. More importantly, our embracement of objective rationality indicates that a trans-mystical mindset may add logic, validity, and scientific credence to the study of metaphysical contexts and mystical experiences. In other words, from our point of view, a trans-mystical mindset may serve as a theoretical lens, helping society and individuals to view the subject matters of metaphysicism objectively. That the metaphysical concept of premonition (Cameron, 1958; Dossey, 2009) is non-mystical and/or is just a “norm” that some individuals may experience. We rationalize this position by considering an interesting benchmark or a point of equivalency – namely:

That a trans-mystical mindset, as extraordinary and higher-order, may equate to the *humanistic state of transcendence*.

#### 3.3.1 Point of equivalency for consideration

The underlying account of our rationalization (i.e., a trans-mystical mindset ≈ state of transcendence) is that both a trans-mystical mindset and a state of transcendence are non-ordinary life states and/or non-everyday contexts (Figure 1). For example, a student’s state of self-transcendence is somewhat different from her state of intrinsic motivation for mathematics learning, and/or that the personal context of bushwalking on a Saturday morning does not coincide with a teenager’s trans-mystical mindset. In terms of transcendence, there are numerous theoretical accounts [Reed’s (1991) Self-Transcendence Theory] within the transpersonal psychology literature, but generally speaking, a popular account is from Maslow (1971), which states the following:

Transcendence refers to the very highest and most inclusive or holistic levels of human consciousness, behaving and relating, as ends rather than means, to oneself, to significant others, to human beings in general, to other species, to nature, and to the cosmos” (p. 269).

An analysis of Maslow’s (1971) description of transcendence (e.g., self-transcendence) suggests, perhaps, a very concise, direct, and clear explanation (e.g., that a person’s state of self-transcendence indicates his complex state of consciousness…). At a deeper level, however, we note a few notable keywords or phrases that are somewhat complex – for example: “holistic levels of human consciousness”, “ends rather than means”, and “the cosmos”. We contend that these keywords or phrases serve to support our earlier mentioning: that the underlying nature of transcendence, similar to a trans-mystical mindset, is something that is higher-order and that a lay person does not necessarily experience on a daily basis.

There is research development that has, to date, explored the impact of transcendence. For example, a number of researchers have studied the underlying nature of “self-transcendence” (Conn, 1998; Ruschmann, 2011; Llanos and Martínez Verduzco, 2022), which concerns a person’s ability to transcend beyond her perceived sense of self and, in the process, recognizing that there are elements in life (e.g., nature, social relationship, the universe, divine power, etc.) that constitute the notion of “whole” (e.g., the “wholeness” of a person consist of…). A person’s self-transcendence experience, in this case, showcases her deep understanding and appreciation that there are, perhaps, greater “powers” in life (e.g., a teenager’s perceived spiritual connectedness with God). As such then, this brief theoretical account supports our earlier mentioning regarding the significance and/or the intricacy of a state of self-transcendence: that it is an experience of a higher-order where some of us in society are fortunate or have been fortunate to have encountered.

Again, reiterating our earlier discussion, a state of self-transcendence is higher-order (Maslow, 1969, 1971) but it does not mean that such encounter and/or experience is mystical in any shape or form. It is a psychological state that we purport may adhere to and/or equate to what we term as “transformation” or the “process of transformation” (Figure 1). Transformation for us, in this case, relates to the “transformation” of a person’s “ordinary” state of consciousness (i.e., ordinary mindset) to a more “complex” state of consciousness (i.e., a trans-mystical mindset). In other words, our conceptualization is as follows:

That transformation of a person’s contextual mindset (i.e., ordinary mindset → trans-mystical mindset) equates to or is analogous to a state of self-transcendence, helping her to rationalize, understand, and/or appreciate the nature of metaphysical contexts, mystical experiences, and the like.

The significance of the aforementioning lies in our attempt to objectively rationalize the nature of metaphysical contexts by equating the concept of a trans-mystical mindset with a state of self-transcendence (Maslow, 1969, 1971). In this analysis, a trans-mystical mindset is not some unknown, mysterious concept that only a few of us may experience. Rather, equating to a state of self-transcendence, a person’s transformed mindset (i.e., personal mindset → trans-mystical mindset) espouses his intimate sense and/or experience of quality attributes, such as *awareness*, *realization*, *logical reasoning*, *acceptance*, and *enlightenment*. As an example, consider a senior citizen who recently encounters a metaphysical life context (e.g., interaction with perceived dark energy of loved ones who have moved on). Such a metaphysical encounter could potentially “transform” the senior citizen’s mindset (i.e., personal mindset → trans-mystical mindset) to assist him to logically rationalize (e.g., he reasons that his experience of spiritual connection is normal), realize (e.g., he realizes that he is able to “sense” a loved one who has moved on nearby), and/or accept (e.g., he accepts that what he is feeling (i.e., sensing a spiritual connection) is normal) that his personal experience of mysticism is normal.

### 3.4 Innovation and intricacy

Figure 1 encapsulates our conceptualization, showcasing the process of transformation and the two major levels of human existence and/or human psyche (i.e., ordinary_(Level 1)_ → trans-mystical_(Level 2)_). Innovatively and significantly, our conceptualization is intended to support and/or to accentuate our theoretical position: that the subject matters of metaphysicism (e.g., a teenager’s mystical experience) are, in fact, “normal” or that they coincide with the realm of objective rationalization. That we may, in fact, use psychological premises (e.g., the use of philosophical psychology) to decipher, normalize, and scientize the perceived “extraordinary” nature of metaphysical context, mystical experiences, and the like. By all accounts, one may perceive and view the context of premonition (Cameron, 1958; Dossey, 2009) as being something that is extraordinary and situates outside or beyond the realm of ordinary boundaries of life and death. This standing, however, emphasizes the importance of subjective experience (e.g., something that is perceived and viewed as being “extraordinary” for one person may not be so for another person). Moreover, such differences in personal experience may make the same belief subjectively rational for one person but not another person. Upon reflection though, we offer an alternative account, which is illustrated here in this section, where we contend that variations in mystical or metaphysical contexts may “cross-reference” with Maslow’s (1969, 1971) hierarchy of needs framework:

**Level 1: an ordinary mindset:** Ordinary boundaries of human existence and/or human psyche may give rise to the proposition of a person’s “ordinary mindset”. Ordinary boundaries of human existence and/or human psyche (e.g., a student’s love for mastery of classical music), from our rationalization, coincide with Maslow’s (1968, 1969) proposition of physiological needs, safety needs, belonging and love needs, and esteem needs. Level 1, from our point of view, is considered as a basic level or a low level of human psyche.

**Level 2: a trans-mystical mindset:** Extraordinary boundaries of human existence and/or human psyche may give rise to the proposition of a “trans-mystical mindset”. Extraordinary boundaries of human existence and/or human psyche (e.g., a teenager’s perceived ability to transcend to another time–space realm), from our rationalization, coincide with Maslow’s (1968, 1969) proposition of self-actualization and self-transcendence. Level 2, from our point of view, is considered as a complex level or a higher level of human psyche.

Our philosophization, summarized in Figure 1, is innovative for its proposition of an active process of transformation of a person’s psychological mindset. That a person’s mindset is contextual (Phan et al., 2024) and changes with reference to a specific context at hand (i.e., Level 1 → L2). Moreover, from our point of view, normalizing and/or scientizing the subject matters of metaphysicism, mysticism, and the like may consist of the equivalency between two higher-order concepts: a trans-mystical mindset ≈ a state of self-transcendence. Variations in human experiences, ranging from ordinary and perceived realistic levels (e.g., one’s personal desire to live a cherished and self-fulfilling life) to extraordinary and perceived complex levels (e.g., one’s personal desire to seek theoretical understanding of the unknowns) may serve to change one’s psychological mindset (i.e., personal mindset → trans-mystical mindset).

## 4 Importance of antecedents: life wisdom and historical-sociocultural contextualization

Approaching the study of life and death education from a mystical perspective (Phan et al., 2021, 2023), or from the perspective objectivity vs. subjectivity, is insightful and interesting, as it may help advance theoretical understanding of the subject matters. An important issue for consideration, in this case, relates to one’s inclination to *accept* or to *reject* the enigma of the subject of trans-mysticism (e.g., a person’s perceived mystical life experience, such as his ability to transcend to another time–space context). Our attempt over the past few years has involved the use of philosophical analysis to help normalize the subject matter of mystical experiences and metaphysical contexts. That psychological premises, for example, may enable us to scientize the nature of metaphysicism. Interestingly, one of our reviewers recently mentioned a pivotal point, contending that philosophizing the relevance and/or the uniqueness of mystical experiences and metaphysical contexts does necessarily make them any more valid. That a person’s willingness to embrace the subject of trans-mysticism, likewise, may simply reflect and/or indicate his sense of curiosity, interest, etc. and nothing more. If this is the case, then it may be plausible to purport that universal contexts (e.g., the context of mastery and enjoyment of visual arts vs. the context of reincarnation) do not conjecture any “mystique” or “extraordinariness”. A specific life context is only mysterious or extraordinary (e.g., a teenager’s conviction that her personal experience of spiritual connection with a loved one who has moved on), perhaps, from a subjective point of view. Having said this, however, we want to briefly introduce two theoretical concepts that may offer grounding and discount the objective logic, validity, and/or legitimacy of trans-mysticism, metaphysical contexts, and the like:

- The importance of life wisdom.
- The importance of historical-sociocultural contextualization.

To offer a balanced overview and to encourage scholarly dialogues, we have chosen to consider an alternative and/or a related viewpoint: that acquired life wisdom and/or one’s historical-sociocultural upbringing may predominate and support and/or strengthen the perspective of subjective rationality. This viewpoint considers the importance of subjectivity, personal experience and interpretation, and individual differences and contends that perhaps there *is* something mysterious about the study of metaphysicism. For example, life wisdom is an interesting commodity that may impart contextual epistemological beliefs, expectations, reflective thoughts, and the like. In a similar vein, historical-sociocultural grounding and/or upbringing may cultivate the cultural belief that ancestor worshipping (Steadman et al., 1996; Lakos, 2010) enables a person to engage in spirit communication.

### 4.1 The importance of life wisdom

*Life wisdom* or life knowledge is somewhat different from contextual subject knowledge (e.g., knowledge of Algebra) as it connotes the importance of “generality”. Situating within the context of life and death education (Chen, 2013; Huang, 2014; Seng and Lee, 2022), life wisdom is defined as:

“A lifelong process that reflects cognitive maturity, diverse life experiences, and the continuation of acquired knowledge of different contexts. A person's wisdom of life, in this sense, is not analogous with his/her intellectual or cognitive development” (Phan et al., 2021).

Unlike specific content knowledge, procedural knowledge, and/or conceptual knowledge (e.g., Algebra), life knowledge, or life wisdom, is somewhat generic and reflects a person’s maturity and diverse life experiences (e.g., a Buddhist nun’s life knowledge of spirituality). Progress in life, in this sense, may coincide with a person’s acquirement and/or development of life knowledge. It is interesting to note life and death education teaching considers the importance of “active transformation” of life wisdom, or life knowledge, into practice for positive and/or effective life functioning (Phan et al., 2021, 2023). Active transformation, importantly, emphasizes the nexus between theory and practical purposes. In terms of the present context, however, we posit that life wisdom may help to assist a person to view metaphysical contexts and mystical experiences somewhat differently. In other words, resonating with our earlier mentioning, a person’s life wisdom may in fact assist him with his subjective interpretation and rationalization – that, indeed, there is logic to the argument that metaphysical cases of reincarnation, premonition, spirit communication, etc. are extraordinary and situate outside the realm of ordinary boundaries of life and death.

### 4.2 The importance of historical-sociocultural and ethno-anthropological contextualization

*Historical-sociocultural background* and *upbringing* (e.g., a South African child who was born and grows up in Indonesia) may help to shape a person’s epistemological belief, cultural value, customary practice, etc. Extensive research development, to date, has acknowledged the importance of what is known as “sociocultural contextualization” or “situational placement” of one’s learning experiences and personal development (Wertsch et al., 1995; Kozulin, 1999; Mahn, 1999). There are specific examples, briefly introduced here, that support the potency of historical-sociocultural and ethno-anthropological premises of life and death experiences. That a person’s specific historical-sociocultural upbringing may play a prominent role, helping to convince her that subjective, metaphysical, and extraordinary contexts are perhaps logical. For example, unlike their Western counterparts, Tibetans in general have been brought up from an early age to appreciate the importance of Tibetan Buddhist teaching (Lama and Chodron, 2019, Prude, 2019), which emphasizes the premise of reincarnation (Burley, 2014; Barua, 2017) or the notion of the “birth-death-rebirth” cycle (Park, 2014; Sarao, 2017). It is their collective cultural belief perhaps, that upon death, one would reincarnate to a new “being” or a new life. In a similar vein, as we cited earlier, many Taiwanese believe in what is known as an “underworld”, or a place where one could meet and communicate with loved ones who have moved on (Phan et al., 2021). It is interesting to note though, that some Western scholars (Greber, 1979; Buckland, 2004; Tymn, 2014; Pócs, 2019) have also made reference to the notion of “spirit communication”.

The brief accounts, as mentioned here, emphasize the potential relevance and applicability of personal upbringing, grounded in historical-sociocultural contexts. Similar to the case of life wisdom, we posit that historical-sociocultural contexts may support the theoretical lens of subjective rationalization. That a particular historical-sociocultural grounding may instill conviction, personal resolve, and/or firm belief that metaphysical encounters, mystical contexts, and the like are ontologically subjective not rational in perception, interpretation, etc.

## 5 Summation

In summation, the study of life and death education (Chen, 2013; Huang, 2014; Seng and Lee, 2022) has established strong grounding for learning, research, and practical purposes. Central to this thesis is a pervasive desire for individuals to appreciate life and death experiences in all different forms. Philosophical, conceptual, and empirical research undertakings have been plentiful, resulting in a myriad of findings and viewpoints for consideration. Our own research inquiries of life and death education over the years, likewise, have provided some interesting findings and insights for continuing teaching and research development. One particular aspect for continuing development relates to the context of universality. Do all of us view, perceive, and/or interpret universal contexts the same or differently? That perhaps, for some of us, life and death contexts are different and exist outside or beyond the ordinary and realistic boundaries of humankind (e.g., a person’s perceived feeling and/or experience of time–space transportation). Indeed, as a recap, we have briefly explored this metaphysical or mystical topic of human agency in a few of our recent articles. This concerted effort has provided preliminary grounding for our proposition of a related psychological concept known as “trans-mysticism”.

The present article considers an interesting discourse: that we may, in fact, subsume and/or frame different subjective viewpoints and interpretations of universal contexts within one common objective, psychological lens. That a resulting trans-mystical mindset, in this case, may help to “objectivize” or scientize the subject matters of metaphysicism, mysticism, and the like. Relating to this proposition is our conceptualization of an equivalency between the process of transformation of an ordinary mindset and a personal state of self-transcendence (i.e., a trans-mystical mindset ≈ a state of self-transcendence). Our philosophization (e.g., situating the concept of a “trans-mystical mindset” within Maslow’s (1968, 1969) hierarchy of needs framework), in this analysis, is intended to achieve three major feats:

- To promote the possibility of normalization and acceptance of metaphysical contexts and mystical life and death experiences from the perspective of psychology.
- To introduce an alternative nomenclature or psychological concept, known as trans-mysticism, into mainstream trans-humanistic literatures for consideration – for example, a person’s contextual mindset may situate within a hierarchy, transforming from an ordinary level to a higher-order level or a trans-mystical level.
- To advance the study of life and death education by considering the legitimacy, logic, and validity of non-conventional or non-objective themes (e.g., the personal experience of premonition).

Overall, then, the focus of our philosophical inquiry raises several notable issues for consideration and/or acknowledgment. That innovatively and creatively, we have utilized psychological premises (e.g., the study of transpersonalism) and the formal teaching and research of life and death education (Chen, 2013, Huang, 2014, Seng and Lee, 2022) to normalize and/or to scientize the subject matters of metaphysicism. Equally important is a focus that we briefly mentioned for future development, which seeks to elucidate the epistemic legitimacy or validation of personal conviction and belief that metaphysical contexts and/or mystical experiences are truly unique [e.g., is there something truly unique, objectively, about one’s mystical belief of a metaphysical encounter (e.g., his conviction that spirit communication is unique and does not coincide with everyday objective reality?)].

## 6 Inquiries for consideration: teaching, educational, and practical purposes

We acknowledge that it is somewhat difficult to conceptualize concretely the concept of trans-mysticism, and/or to convince someone that there is scientific truth to the subject matters of metaphysicism and mysticism. Unlike other theories, concepts, relationships, etc. in the social sciences (e.g., the study of *human motivation* for effective learning), trans-humanism in its entirety is somewhat abstract, subjective, and individualized, requiring philosophical analysis, reasoned judgment, and contemplation to assist with the attainment of meaningful understanding. In this section of the article, we introduce a few proposed inquiries that may add valuable insights and support our aforementioned proposition for further development.

### 6.1 Teaching and practical purposes

Quality teaching (e.g., on-campus) and innovative curriculum development, as a whole, is a central element of successful schooling and academic learning experiences. The nexus between research and learning outcomes may involve active transformation of research findings into practice, where possible [e.g., how do we transform the premise of premonition (Cameron, 1958, González-González, 2019) into positive daily practice?]. Our interest in this matter over the past few years has been to develop a “unifying” framework of life and death education that may take into account different theoretical lenses – *psychological*, *philosophical*, *sociological*, *anthropological*, etc. Such a unifying framework could, perhaps, help to provide complementary information for holistic understanding of the subject contents of life and death [e.g., a psychological viewpoint (e.g., psychological process of grief) + historical-sociocultural viewpoint (e.g., the Eastern viewpoint about death) of death].

Aside from a unifying framework that incorporates different theoretical lenses, what else can we consider for effective teaching and learning experiences? Consider, in this case, innovative curriculum development that places emphasis on daily relevance and applied educational and non-educational practices. Does a trans-mystical mindset have any practicality for consideration? Can a student utilize her trans-mystical life experience or an encountered metaphysical context to “better” herself and/or others? Is there a program for implementation that an educator could develop, which takes into account the importance of trans-mystical life/death contexts? These sample questions emphasize the importance of *practicality* or the *transformation* of theory into practice. To answer such questions, we would need to consider the potential negative perception of the subject matter itself – that:

- Some or many students, in general, may not appreciate and/or view trans-mystical life/death contexts as a credible subject for studying (e.g., for their future study and/or career pathways).
- It is somewhat difficult to associate trans-mystical life/death contexts with everyday relevance and/or applicability.
- Some or many students may have differing viewpoints, religious faiths, cultural beliefs, etc. that would prevent them from embracing the subject of trans-mystical life/death contexts.

Mathematics, Biology, Chemistry, Economics, etc. are “hard pure theoretical” disciplines (Becher, 1989; Becher, 1994) that are concrete, relatively straightforward in terms of comprehension, processing, and/or understanding, and may reflect daily life relevance. Where does the subject of metaphysical contexts and/or the subject of mystical experiences, in contrast, rank in terms of “intellectual categorization” (e.g., is there any “academic basis” to the study of trans-mysticism?) (Becher, 1989, 1994)? Becher’s (1989, 1994) *framework of intellectual categorizations* (e.g., treating the subject content of a trans-mystical mindset as a “soft pure theoretical” subject), in this case, may help to define or redefine the “intellectual rigor” of the subject matters of trans-mysticism, metaphysical contexts, and the like. In a similar vein, the pedagogical practice of theoretical infusion (Phan et al., 2023, 2024), as described earlier, may lend support and strengthen the perception of intellectual or “academic rigor” to the subject matters of trans-mysticism, metaphysical contexts, and the like. For example, the pedagogical practice of Buddhist infusion (Yeshe and Rinpoche, 1976; Metzner, 1996; Master Sheng Yen, 2010) may associate trans-mysticism with the subject matter of Buddhist spirituality (e.g., that personal experience and/or feeling of Buddhist spirituality is non-ordinary or extraordinary, reflecting the uniqueness of mysticism), adding valuable academic insights for consideration.

The study of trans-mysticism, in its entirety (e.g., a trans-mystical mindset), may impart some relevant insights for daily life purposes. Daily life relevance, in this case, does not necessarily equate to useful practicalities for positive life functioning. Rather, from our point of view, life relevance arising from in-depth knowledge and personal understanding of trans-mysticism may relate to one’s ability to *appreciate* and accept the broad “humanistic” nature of life and death. Furthermore, appreciating the concept of trans-mysticism may enable and/or assist a person to recognize that interpretation of life and death can incorporate and involve different theoretical lenses – for example, objective reality vs. individual subjectivity.

### 6.2 Self-reflection and holistic state of consciousness-subconsciousness

We now turn our attention to another focus of inquiry, which seeks to consider the potential impact of a person’s trans-mystical mindset on her state of personal reflection. *Personal reflection*, as Schön (1983, 1987) contends, may espouse two different types: “in-action” reflection (i.e., during the event) and “on-action” reflection (i.e., after the event). This theoretical premise is relevant and may, in fact, relate to the context of our discussion of trans-mysticism. There are a few inquiries that we have formulated for researchers, educators, etc. to consider:

- Does a trans-mystical mindset coincide with or help a person to develop reflective thinking skills?
- Does an encounter with a particular trans-mystical context and/or mystical life/death experience help a person to develop reflective thinking skills?
- Can personal reflection assist a person to reason, accept, and/or embrace trans-mystical life/death contexts?
- Can reflective thinking serve as an informational source, helping to necessitate, prepare, facilitate, and/or sustain a trans-mystical mindset?

The main issue, from our point of view, is whether trans-mystical mindset and reflective practice are interrelated with each other. In terms of life and death contexts, specifically, we prefer to use the term “self-contemplation” or “personal contemplation” (Chattopadhyay, 2022) over that of self-reflection. For us, self-contemplation is more than just a state of personal reflection of different types of life functioning. Rather, self-contemplation is transpersonal and reflects a person’s concerted *introspection* to seek deep understanding about life experiences and the true meaning of higher-order life attainments. Moreover, from our point of view, self-contemplation emphasizes the importance of one’s own self-analysis and philosophization about the true meaning of aestheticism and altruism. It would be an interesting endeavor to explore the self-contemplative nature of trans-mysticism. To facilitate this line of questioning, we propose a term that we coin as “trans-mystical contemplation” or “trans-mystical introspection” – for example: does a person’s experience of trans-mysticism (e.g., a person’s conviction and belief that she is able to connect spiritually with loved ones who have moved on) reflect his contemplative or introspective thoughts?

**Figure 2:**
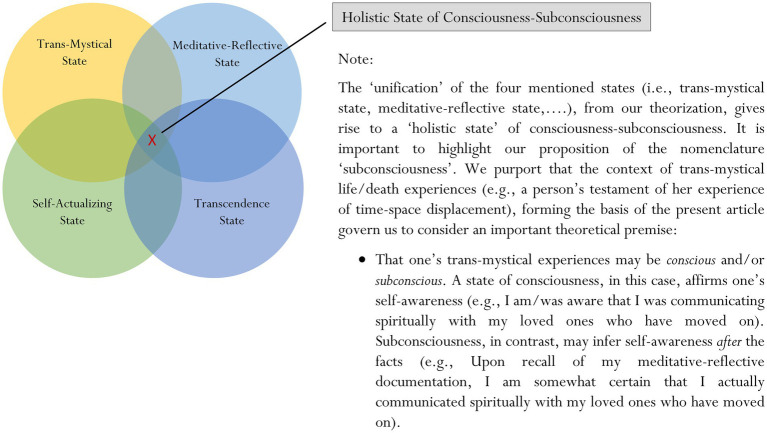
A proposed holistic state of consciousness-subconsciousness here.

Our recent article introduced a mindfulness-related methodological approach known as “meditative-reflective documentation” (Phan et al., 2024). Meditative-reflective documentation is an approach that encourages a person to document and note down specific phrases, drawings, keywords, etc. that could describe his “meditative-reflective” experience. This theoretical account of meditative-reflective experience contends that in-depth meditation may enable a person to attain and/or to experience a higher-order “meditative-reflective” state – for example, his perceived feeling of “extraordinariness”, such as the perceived feeling of out-of-body experience (e.g., self-awareness of the perception of “disassociation” of body and mind from the present time–space context). As a result of this mentioning, we wonder whether there is credence to consider an interesting proposition: that the totality of a person’s state of consciousness and subconsciousness may consist of a unification or a combination of similar states: a *trans-mystical state*, a *meditative-reflective state*, a *self-actualizing state*, a *transcendence state*, etc., (Figure 2).

Our seminal idea, as described, considers the possibility that some discourse and/or course of action could act to facilitate the *unification* of different states of consciousness and subconsciousness (e.g., a trans-mystical state, a meditative-reflective state, a self-actualizing state, and a transcendence state). This unification, we philosophize, may serve to encapsulate the “entirety” of a person’s state of consciousness-subconsciousness. Our narrative, in this case, contends that metaphysical contexts and/or mystical experiences may help initiate, unify, and sustain the four aforementioned states of consciousness-subconsciousness.

### 6.3 Research development for consideration

In this final section of the article, we discuss a few research propositions that may assist to support our advocation for the study of the entirety of trans-humanism, including the proposed concept of a trans-mystical mindset. We acknowledge that overall, the subject of trans-humanism is abstract, philosophical, and can be somewhat incomprehensible at times, making it difficult for students, individuals, etc. to understand and appreciate. Even more difficult, perhaps, is the development of research undertakings that could in effect help to validate such representation(s). There are a couple of questions, at present, for us to consider:

- How do we accurately measure and assess the underlying nature of a trans-mystical mindset?
- How do we measure, assess, and/or evaluate one’s perceived feeling of a metaphysical context, mystical experience, and the like?
- How do we validate the proposition of a holistic state of consciousness-subconsciousness (Figure 2), which may consider the following: a trans-mystical state, a meditative-reflective state, a self-actualizing state, a transcendence state, etc.?
- How do we objectively validate, legitimize, and/or confirm that a trans-mystical mindset is unique or that metaphysical experiences are extraordinary and situate outside the realm of ordinary boundaries?

The sample questions above illustrate the complexities of the study of the entirety of trans-humanism. For example, how would we soundly and/or accurately undertake a research inquiry into the nature of a trans-mystical mindset? This question places emphasis on a research-related issue or matter known as “methodological appropriateness” (Esterberg, 2002; Creswell, 2003; Creswell, 2008). Methodological appropriateness, in brief, relates to the development of an appropriate methodological design for usage that would, in turn, enable a researcher to measure and assess a concept, phenomenon, relationship, etc. adequately and accurately In the social sciences, there are a couple of robust and stringent methodological designs for researchers, educators, students, etc. to consider (e.g., Likert-scale inventories, surveys, open-ended interviews). Likert-scale inventories and/or open-ended surveys are relatively straightforward and, in this case, may offer simple, direct opportunities and/or pathways for the attainment of evidence into the perception of trans-mystical life/death experiences (e.g., I perceive that there is something out there, divine, that I cannot explain…).

An important line of inquiry for consideration entails a comparative analysis of viewpoints, perspectives, interpretations, opinions, etc. of the study of trans-humanism in its entirety. We purport that a “sociocultural-anthropological” approach could offer a more interesting account of perception, interpretation, understanding, etc. of metaphysical contexts, mystical experiences, and the like. A sociocultural-anthropological approach (Phan et al., 2024), we contend, places emphasis on the importance of diverse customary practices, cultural values, epistemological beliefs, protocols, etc. As we mentioned earlier, historical-sociocultural grounding and personal upbringing may play a prominent role, helping to shape or influence a person’s behavior, viewpoint, interpretation, epistemological belief, etc. (e.g., that there is logic and relevance to the cultural practice of ancestor worshipping). In this analysis, research undertakings that place emphasis on ethnographic-anthropological differences or similarities (e.g., the contrasting viewpoints regarding a trans-mystical mindset in reception, belief, and conviction towards the notion of premonition) may lend support for a wider scope in study of perspectives, beliefs, opinions, and ideas of metaphysical contexts, etc.

## 7 Conclusion

The present conceptual analysis article, we contend, has advanced the study of transpersonalism in its entirety (Maslow, 1969; Strohl, 1998; Hartelius et al., 2007; Lancaster and Linders, 2019) by considering an alternative – namely, the nomenclature “trans-humanism” and, in this case, the philosophized psychological concept of trans-mysticism. Our focus of inquiry, philosophically and theoretically, attempts to analyze the potential relevance and significance of trans-mysticism by situating its nature within the framework of life and death education (Phan et al., 2021; Lei et al., 2022; Seng and Lee, 2022; Shu et al., 2023). Specifically, we purport that metaphysical contexts, mystical experiences, and the like may transform a person’s ordinary mindset to a trans-mystical mindset, helping him to appreciate, rationalize, and make reasoned judgments about the nature of such “extraordinary” encounters.

Overall, then, we contend that our focus of inquiry has added valuable insights for research, teaching, and practical purposes. Central to this thesis is our use of philosophical analysis to normalize and scientize a subject area that is perceived as being somewhat non-conventional. This utilization of personal philosophization has provided grounding for consideration of several interesting endeavors: (i) viewing life and death from contrasting theoretical lenses (e.g., objective reality vs. individual subjectivity), (ii) seeking to engage in higher-order human practices (e.g., meditative-reflection) in order to encounter and/or to experience metaphysical contexts and the like, and (iii) embracing the importance of “normalization” of extraordinary human psyche for daily functioning.

## Author contributions

HP: Conceptualization, Formal analysis, Investigation, Methodology, Project administration, Supervision, Validation, Writing – original draft, Writing – review & editing. BN: Conceptualization, Formal analysis, Investigation, Methodology, Validation, Writing – original draft, Writing – review & editing. C-SH: Conceptualization, Investigation, Methodology, Project administration, Resources, Validation, Writing – review & editing. S-CC: Conceptualization, Funding acquisition, Investigation, Methodology, Project administration, Resources, Supervision, Writing – review & editing. LW: Conceptualization, Funding acquisition, Investigation, Methodology, Resources, Writing – review & editing.

## Footnotes

^1^ https://www.theguardian.com/lifeandstyle/2021/oct/23/premonitions-that-turn-out-to-be-true-is-there-science-to-explain-them-amelia-tait

## References

[ref1] Barua A. (2017). The reality and the verifiability of reincarnation. Religions 8, 1–13. doi: 10.3390/rel8090162

[ref2] Becher T. (1989). Academic Tribes and Territories. Milton Keynes, UK, Open University Press.

[ref3] Becher T. (1994). The significance of disciplinary differences. Stud. High. Educ. 19, 151–161. doi: 10.1080/03075079412331382007, PMID: 38594599

[ref4] Bronkhorst J. (2022). Mystical experience. Religions 13, 1–20. doi: 10.3390/rel13070589

[ref5] Buckland R. (2004). Buckland's Book of Spirit Communications. St. Paul, Minnesota. Llewellyn Publications.

[ref6] Burley M. (2014). Taking reincarnation seriously: critical discussion of some central ideas from John hick. Int. J. Philos. Theol. 75, 236–253. doi: 10.1080/21692327.2014.967975

[ref7] Cameron A. (1958). Premonition of death. Br. Med. J. 2:914. doi: 10.1136/bmj.2.5101.914-a

[ref8] Chapple H. S. Bouton B. L. Chow A. Y. M. Gilbert K. R. Kosminsky P. Moore J.. (2017). The body of knowledge in thanatology: an outline. Death Stud. 41, 118–125. doi: 10.1080/07481187.2016.1231000 27611636

[ref9] Chattopadhyay M. (2022). Contemplation: its cultivation and culmination through the Buddhist glasses. Front. Psychol. 12:281. doi: 10.3389/fpsyg.2021.800281, PMID: 35449693 PMC9017814

[ref10] Chen S.-C. (2013). Overview and reflection on the 20-year National Education Life Education Curriculum. Natl Educ. 53, 1–6.

[ref11] Clark C. S. (2016). Watson's human caring theory: pertinent transpersonal and humanities concepts for educators. Humanities 5, 1–12. doi: 10.3390/h5020021

[ref12] Clark K. J. Palmer C. T. (2016). “Ancestor worship” in Encyclopedia of Evolutionary Psychological Science. eds. Weekes-Shackelford V. Shackelford T. K. Weekes-Shackelford V. A. (Cham: Springer International Publishing), 1–3.

[ref13] Cohen-Zimerman S. Cristofori I. Zhong W. Bulbulia J. Krueger F. Gordon B.. (2020). Neural underpinning of a personal relationship with god and sense of control: a lesion-mapping study. Cogn. Affect. Behav. Neurosci. 20, 575–587. doi: 10.3758/s13415-020-00787-4, PMID: 32333240

[ref14] Conn W. E. (1998). Self-transcendence, the true self, and self-love. Pastor. Psychol. 46, 323–332. doi: 10.1023/A:1023063820862

[ref15] Creswell J. W. (2003). Research Design: Qualitative, Quantitative, and Mixed Methods Approaches. Thousand Oaks, CA, Sage Publications, Inc.

[ref16] Creswell J. W. (2008). Educational Research: Planning, Conducting, and Evaluating Quantitative and Qualitative Research. Upper Saddle River, N.J.: Pearson/Merrill Prentice Hall.

[ref17] Cunningham P. F. (2007). The challenges, prospects, and promise of transpersonal psychology. Int. J. Transpers. Stud. 26, 41–55. doi: 10.24972/ijts.2007.26.1.41

[ref18] Daniels M. (2002). The transpersonal self: a psychohistory and phenomenology of the soul. Transpers. Psychol. Rev. 6, 17–28.

[ref19] Dossey L. (2009). Extended human capacities: the power. Shift Front. Conscious. 23, 12–17.

[ref20] Esterberg K. G. (2002). Qualitative Methods in Social Research. New York, NY, McGraw Hill.

[ref21] Friedman H. (2002). Transpersonal psychology as a scientific field. Int. J. Transpers. Stud. 21, 175–187. doi: 10.24972/ijts.2002.21.1.175, PMID: 31311435

[ref22] Ge B. H. Yang F. (2023). Transcending the self to transcend suffering. Front. Psychol. 14:1113965. doi: 10.3389/fpsyg.2023.1113965, PMID: 37484086 PMC10361767

[ref23] Goldstein J. Kornfield J. (1987). Seeking the Heart of Wisdom: The Path of Insight Meditation. Boston, MA, Shambhala Publications, Inc.

[ref24] González-González J. M. (2019). Physical theory of premonition in medicine. Int. J. Sci. Res. 8, 1340–1344.

[ref25] Greber J. (1979). Communication with the Spirit World of God: Its Laws and Purpose, Extraordinary Experiences of a Catholic Priest. Teaneck, NJ, Johannes Greber Memorial Foundation.

[ref26] Hanfstingl B. (2022). Future objectivity requires perspective and forward combinatorial meta-analyses. Front. Psychol. 13:908311. doi: 10.3389/fpsyg.2022.908311 35783689 PMC9247499

[ref27] Hartelius G. Caplan M. Rardin M. A. (2007). Transpersonal psychology: defining the past, divining the future. Humanist. Psychol. 35, 135–160. doi: 10.1080/08873260701274017

[ref28] Havens T. (2013). Confucianism as humanism. CLA J. 1, 33–41.

[ref29] Huang J. (2014). New Orientation of Life Education in the 21st Century: Spiritual Awakening, Classic Study and Environmental Education. Proceedings of the Ninth Life Education Conference, Taipei City, Taiwan, Taiwan Life Education Society.

[ref30] Jones R. (2016). Mysteries of the Afterlife. Eugene, Oregon, Harvest House Publishers.

[ref31] Kozulin A. (1999). Sociocultural contexts of cognitive theory. Hum. Dev. 42, 78–82. doi: 10.1159/000022612, PMID: 38557347

[ref32] Lajoie D. H. Shapiro S. I. (1992). Definitions of transpersonal psychology: the first twenty-three years. J. Transpers. Psychol. 24, 79–98.

[ref33] Lakos W. (2010). Chinese Ancestor Worship: A Practice and Ritual Oriented Approach to Understanding Chinese Culture. Newcastle upon Tyn, UK, Cambridge Scholars Publishing.

[ref34] Lama Dalai Chodron T. (2019). Samsara, Nirvana, and Buddha Nature. Somerville, MA, Wisdom Publications.

[ref35] Lancaster B. L. Linders E. H. (2019). “Spirituality and transpersonalism” in The Routledge International Handbook of Spirituality in Society and the Professions. eds. Zsolnai L. Flanagan B. (New York, NY, US: Routledge/Taylor & Francis Group), 40–47.

[ref36] Laurin K. Schumann K. Holmes J. G. (2014). A relationship with god? Connecting with the divine to assuage fears of interpersonal rejection. Soc. Psychol. Personal. Sci. 5, 777–785. doi: 10.1177/1948550614531800

[ref37] Lei L. Lu Y. Zhao H. Tan J. Luo Y. (2022). Construction of life-and-death education contents for the elderly: a Delphi study. BMC Public Health 22:802. doi: 10.1186/s12889-022-13197-7, PMID: 35449042 PMC9022733

[ref38] Llanos L. F. Martínez Verduzco L. (2022). From self-transcendence to collective transcendence: in search of the order of hierarchies in Maslow’s transcendence. Front. Psychol. 13, 1–9. doi: 10.3389/fpsyg.2022.787591 PMC8988189 35401301

[ref39] Long J. (2000). Spirituality and the idea of transcendence. Int. J. Child. Spiritual. 5, 147–161. doi: 10.1080/713670913

[ref40] Lundberg A. Fraschini N. Aliani R. (2023). What is subjectivity? Scholarly perspectives on the elephant in the room. Qual. Quant. 57, 4509–4529. doi: 10.1007/s11135-022-01565-9

[ref41] Mahn H. (1999). Vygotsky's methodological contribution to sociocultural theory. Remedial Spec. Educ. 20, 341–350. doi: 10.1177/074193259902000607

[ref42] Masel E. K. Schur S. Watzke H. H. (2012). Life is uncertain. Death is certain. Buddhism and palliative care. J. Pain Symptom Manag. 44, 307–312. doi: 10.1016/j.jpainsymman.2012.02.018, PMID: 22871512

[ref43] Maslow A. H. (1968). Toward a Psychology of Being. Princeton, NJ, Van Nostrand Reinhold.

[ref44] Maslow A. H. (1969). Various meanings of transcendence. J. Transpers. Psychol. 1, 56–66.

[ref45] Maslow A. H. (1971). The Farther Reaches of Human Nature. New York, NY, Arkana/Penguin Books.

[ref46] Master Sheng Yen (2010). The Dharma Drum Lineage of Chan Buddhism: Inheriting the Past and Inspiring the Future. Taipei City, Taiwan, The Sheng Yen Education Foundation.

[ref47] McCartney K. Dearing E. Taylor B. A. Bub K. L. (2007). Quality child care supports the achievement of low-income children: direct and indirect pathways through caregiving and the home environment. J. Appl. Dev. Psychol. 28, 411–426. doi: 10.1016/j.appdev.2007.06.010, PMID: 19578561 PMC2705127

[ref48] McDermott R. A. (1993). “Transpersonal worldviews: historical and philosophical reflections” in Paths Beyond Ego: The Transpersonal Vision. eds. Walsh R. Vaughan F. (Tarcher/Perigee: Los Angeles, US), 206–212.

[ref49] Meagher D. J. Balk D. E., (Eds.) (2013). Handbook of Thanatology. London, UK, Routledge.

[ref50] Metzner R. (1996). The Buddhist six-worlds model of consciousness and reality. J. Transpers. Psychol. 28, 155–166.

[ref51] Mistry R. S. Benner A. D. Tan C. S. Kim S. Y. (2009). Family economic stress and academic well-being among Chinese-American youth: the influence of adolescents' perceptions of economic strain. J. Fam. Psychol. 23, 279–290. doi: 10.1037/a0015403, PMID: 19586191 PMC2761095

[ref52] Nagaraj A. Nanjegowda R. B. Purushothama S. (2013). The mystery of reincarnation. Indian J. Psychiatry 55, 171–176. doi: 10.4103/0019-5545.105519, PMID: 23858250 PMC3705678

[ref53] Park M.-S. K. (2014). Samsara: When, where and in what form shall we meet again? (Master of Fine Arts). University of Sydney, Sydney, Australia. Retrieved from https://ses.library.usyd.edu.au/handle/2123/13441?show=full Available from The University of Sydney Sydney eScholarship database.

[ref54] Pasi M. (2015). “Esoteric experiences and critical ethnocentrism” in Religion: Perspectives from the Engelsberg Seminar 2014. eds. Almqvist K. Linklater A. (Stockholm, Sweden: Axel and Margaret Ax:son Johnsons Foundation), 131–142.

[ref55] Phan H. P. Chen S.-C. Ngu B. H. Hsu C.-S. (2023). Advancing the study of life and death education: theoretical framework and research inquiries for further development. Front. Psychol. 14, 1–13. doi: 10.3389/fpsyg.2023.1212223 PMC10413111 37575440

[ref56] Phan H. P. Ngu B. H. Chen S.-C. Hsu C.-S. (2024). An ideal sense of self: proposition of holistic self and holistic mindset from the unique anthropological-sociocultural perspective of life and death education. J. Theoretical Philos. Psychol., 1–28. doi: 10.1037/teo0000265

[ref57] Phan H. P. Ngu B. H. Chen S.-C. Wu L. Shih J.-H. Shi S.-Y. (2021). Life, death, and spirituality: a conceptual analysis for educational research development. Heliyon 7:e06971. doi: 10.1016/j.heliyon.2021.e06971, PMID: 34036188 PMC8138599

[ref58] Pócs É., Ed. (2019). Body, Soul, and Spirits and Supernatural Communication. Newcastle upon Tyn, UK, Cambridge Scholars Publishing.

[ref59] Prude A. (2019). “Death in Tibetan Buddhism” in Death and Dying: An Exercise in Comparative Philosophy of Religion. eds. Knepper T. D. Bregman L. Gottschalk M., vol. 2 (Cham: Springer International Publishing), 125–142.

[ref60] Reed P. (1991). Toward a nursing theory of self-transcendence: deductive reformulation using developmental theories. Adv. Nurs. Sci. 13, 64–77. doi: 10.1097/00012272-199106000-00008, PMID: 2059006

[ref61] Ruschmann E. (2011). Transcending towards transcendence. Implicit Religion 14, 421–432. doi: 10.1558/imre.v14i4.421, PMID: 38513534

[ref62] Rush M. (2011). The esoteric experience: positive or negative? Paranthropology 2, 3–8.

[ref63] Sarao K. T. S. (2017). “Saṃsāra (Buddhism)” in Buddhism and Jainism. eds. Sarao K. T. S. Long J. D. (Netherlands: Dordrecht, Springer), 1048–1050.

[ref64] Schneiderman L. (1967). Psychological notes on the nature of mystical experience. J. Sci. Study Relig. 6, 91–100. doi: 10.2307/1384201

[ref65] Schneiders S. M. (1986). Theology and spirituality: strangers, rivals, or partners? Horizons 13, 253–274. doi: 10.1017/S036096690003632X, PMID: 38599789

[ref66] Schön D. (1983). The Reflective Practitioner: How Professionals Think in Action. New York, Basic Books.

[ref67] Schön D. (1987). Educating the Reflective Practitioner. San Francisco, CA, Jossey-Bass.

[ref68] Segal A. F. (2004). Life After Death: A History of the Afterlife in Western Religion. New York, Doubleday.

[ref69] Seng H. Z. Lee P. W. (2022). Death education in Malaysia: from challenges to implementation. Int. J. Pract. Teach. Learn. 2, 1–8.

[ref70] Shorrock A. (2008). The Transpersonal in Psychology, Psychotherapy and Counselling. New York, NY, Palgrave MacMillan.

[ref71] Shu W. Miao Q. Feng J. Liang G. Zhang J. Zhang J. (2023). Exploring the needs and barriers for death education in China: getting answers from heart transplant recipients' inner experience of death. Front. Public Health 11:1082979. doi: 10.3389/fpubh.2023.1082979, PMID: 36860384 PMC9968799

[ref72] Steadman L. B. Palmer C. T. Tilley C. F. (1996). The universality of ancestor worship. Ethnology 35, 63–76. doi: 10.2307/3774025, PMID: 27154194

[ref73] Sternberg R. Glück J., (Eds.) (2019). The Cambridge Handbook of Wisdom. Cambridge, Cambridge University Press.

[ref74] Strohl J. E. (1998). Transpersonalism: Ego meets soul. J. Couns. Dev. 76, 397–403. doi: 10.1002/j.1556-6676.1998.tb02698.x, PMID: 34231893

[ref75] Tart C., (Ed.) (1975). Transpersonal Psychologies. London, UK, Routledge and Kegan Paul.

[ref76] Thagard P. (2014). The self as a system of multilevel interacting mechanisms. Philos. Psychol. 27, 145–163. doi: 10.1080/09515089.2012.725715

[ref77] Thagard P. (2018). Mind, consciousness, and free will. Front. Philos. China 13, 377–393. doi: 10.3868/s0300-007-018-0029-2

[ref78] Townsend N. (1969). Ancestor Worship and Social Structure: A Review of Recent Analyses. Master of Arts, McMaster University.

[ref79] Tymn M. E. (2014). Communication with the Spirit world of god: its Laws and Purpose. Extraordinary experiences of a Catholic priest by Johannes Greber. J. Sci. Explorat. 27, 726–734.

[ref80] Villani D. Sorgente A. Iannello P. Antonietti A. (2019). The role of spirituality and religiosity in subjective well-being of individuals with different religious status. Front. Psychol. 10:1525. doi: 10.3389/fpsyg.2019.01525, PMID: 31354566 PMC6630357

[ref81] Wagani R. Colucci E. (2018). Spirituality and wellbeing in the context of a study on suicide prevention in North India. Religions 9, 1–18. doi: 10.3390/rel9060183

[ref82] Wertsch J. V. del Rio P. Alvarez A. (1995). Sociocultural Studies of Mind. Cambridge, Cambridge University Press.

[ref83] Willyard C. (2023). Are repeat COVID infections dangerous? What the science says. Nature 616, 650–652. doi: 10.1038/d41586-023-01371-9, PMID: 37100944

[ref84] Yao X. (2000). An Introduction to Confucianism. Cambridge, UK, Cambridge University Press.

[ref85] Yeshe L. Rinpoche L. Z. (1976). Wisdom Energy: Basic Buddhist Teachings. Somerville, MA, Wisdom Publications.

[ref86] Zhang M. (2003). Links between school absenteeism and child poverty. Pastoral Care Educ. 21, 10–17. doi: 10.1111/1468-0122.00249, PMID: 35666971

